# Survey of Image Processing Techniques for Brain Pathology Diagnosis: Challenges and Opportunities

**DOI:** 10.3389/frobt.2018.00120

**Published:** 2018-11-02

**Authors:** Martin Cenek, Masa Hu, Gerald York, Spencer Dahl

**Affiliations:** ^1^Department of Computer Science, University of Portland, Portland, OR, United States; ^2^TBI Imaging and Research, Alaska Radiology Associates, Anchorage, AK, United States; ^3^Columbia College, Columbia University, New York, NY, United States

**Keywords:** brain, MRI imaging, image processing, traumatic brain injury, tumor detection, machine learning, artificial intelligence, decision support tool

## Abstract

In recent years, a number of new products introduced to the global market combine intelligent robotics, artificial intelligence and smart interfaces to provide powerful tools to support professional decision making. However, while brain disease diagnosis from the brain scan images is supported by imaging robotics, the data analysis to form a medical diagnosis is performed solely by highly trained medical professionals. Recent advances in medical imaging techniques, artificial intelligence, machine learning and computer vision present new opportunities to build intelligent decision support tools to aid the diagnostic process, increase the disease detection accuracy, reduce error, automate the monitoring of patient's recovery, and discover new knowledge about the disease cause and its treatment. This article introduces the topic of medical diagnosis of brain diseases from the MRI based images. We describe existing, multi-modal imaging techniques of the brain's soft tissue and describe in detail how are the resulting images are analyzed by a radiologist to form a diagnosis. Several comparisons between the best results of classifying natural scenes and medical image analysis illustrate the challenges of applying existing image processing techniques to the medical image analysis domain. The survey of medical image processing methods also identified several knowledge gaps, the need for automation of image processing analysis, and the identification of the brain structures in the medical images that differentiate healthy tissue from a pathology. This survey is grounded in the cases of brain tumor analysis and the traumatic brain injury diagnoses, as these two case studies illustrate the vastly different approaches needed to define, extract, and synthesize meaningful information from multiple MRI image sets for a diagnosis. Finally, the article summarizes artificial intelligence frameworks that are built as multi-stage, hybrid, hierarchical information processing work-flows and the benefits of applying these models for medical diagnosis to build intelligent physician's aids with knowledge transparency, expert knowledge embedding, and increased analytical quality.

## 1. Introduction

Medical diagnosis aided by software systems that use a variety of artificial intelligent and machine learning algorithms have been available since early 1990s (Kononenko, 2001). These medical diagnostic tools use a patient's symptoms, diagnostic measurements and lab results as inputs, and the system returns a ranked list of diagnoses along with suggested treatments (Kononenko, 1993; Soni et al., 2011). Although such tools exist to diagnose a general illness, radiology does not yet have a generalized tool which can assist in diagnosis.

Previous research failed to create a tool which would assist in general analysis of radiology images for several reasons that include the lack of (1) transparency of machine learning and artificial intelligence algorithms to provide feedback on which features were detected and used by the algorithm to make its diagnostic decision, (2) large, diverse, annotated, longitudinal, open-source data sets that are needed to build and train the diagnostic algorithms, (3) the massive hardware support necessary to process high fidelity 3D images and extract useful information.

Recent advancements in the non-invasive, soft-tissue imaging modalities is generating large volumes of high resolution data. Radiologists use high resolution soft tissue images to diagnose numerous medical conditions such as brain cancer, aneurysms, traumatic brain injury, and viral infections. Soft tissue scans are rich with feature information that helps the practitioner differentiate healthy tissue from the pathological cases. A variety of specialized imaging techniques is need for diagnosis of a particular brain anomaly and no single imaging technique is sufficient as a general diagnostic imaging (Berquist et al., 1990). However, combining image sets from multiple imaging techniques can create a unique signature for each soft tissue type (Havaei et al., 2017), producing a large number of features for post processing by a machine learning (ML) algorithm. Even a radiology specialist needs several hours to analyse multiple soft-tissue image sets, extract meaningful features, and integrate the partial findings to create a feature composite used to formulate a diagnosis for a single patient. The application of machine learning algorithms in this domain is limited to image pre-processing and analysis of structural brain changes through segmentation. The opportunity to augment the imaging robotics with intelligent image processing can produce a wide range of decision support tools (DST) that will (1) reduce the time it takes for a radiologist to analyse images to extract meaningful features, (2) diagnose brain disease that cannot be detected from the images using naked eye although the information exists in the images and the machine learning algorithms can learn to differentiate the normal cases from the pathology ones, and (3) automate the monitoring of brain tissue recovery.

**Figure 1:**
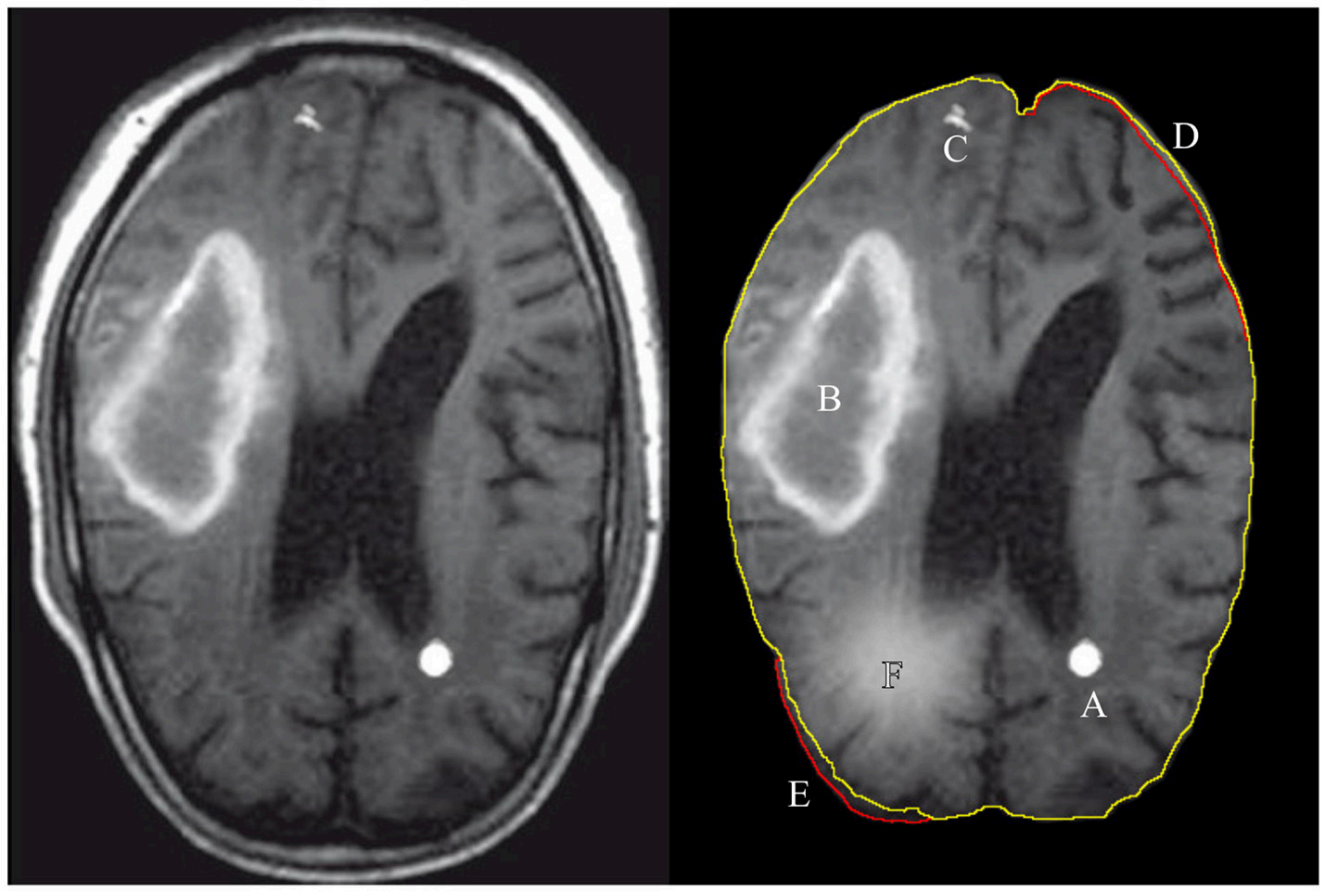
Illustration of features extracted and used by either a radiologist or a computer algorithm. The left images shows the original MRI image, with the annotated features of interest on the right. A: tumor, B: hematoma, C: calcium deposit, D: the volume decrease in the images brain tissue, E: the volume increase with respect to expected and F: a tissue inflammation. The green contour line associated with features D,E shows the expected (normal) shape of the imaged soft tissue and the red contour shows the actual shape.

Radiology professionals are trained to detect the local, regional, and global soft tissue anomalies that deviate from the expected, normal variance in the soft tissue images. Figure 1 shows examples of features that can be categorically described as discrete (A,C) or diffused (B,F) features. Recent studies have shown the use of automated machine learning (ML) algorithms to be successful in diagnosing and segmenting either brain or lung tumors with error rates similar to the human practitioner's error (El-Dahshan et al., 2014; Menze et al., 2016; Shankar et al., 2016; Alakwaa et al., 2017; Havaei et al., 2017). Although a narrow application domain, the use of ML algorithms for feature extraction and classification has significant benefits for the early disease diagnosis that can be easily missed by a physician, malignant tissue segmentation for surgical intervention, and predicting patient's survival rates after forming a preliminary diagnosis.

## 2. Decision support tools' requirements for intelligent robotics

Fusion of new imaging modalities and intelligent image post-processing presents unique opportunities for building a new generation of decision support tools (DST) for brain pathology diagnosis. ML algorithms are well suited to assist in the radiologist's decision making process since they generalize information from a large amount of available data to learn the features and the relationships among the features that differentiate the images in the set between normal and abnormal (Kononenko, 2001). Even in this rudimentary form, ML tools would provide a valuable insight for a physician to confirm and validate the candidate diagnosis selection. However, the transparency of the ML algorithms in making diagnostic decisions is problematic because the exact reasoning used by the algorithm to form the given diagnosis is abstracted during the model construction.

Kononenko proposed general guidelines for a decision support tool that uses ML algorithms for medical diagnostic (Kononenko, 2001):

- Classification performance–a high accuracy of diagnosing the true positive cases while the misdiagnoses are unacceptable (a zero false negative error)
- Noise and variance–the algorithm's ability to extract meaningful information from images with high expected variance or noise introduced during the imaging
- Explanation and transparency of diagnostic knowledge–the ability to trace back the model's decision making and identification of the features it used in the reasoning process.
- Missing data–the ability to fill-in information when the imaging robotics was configured for low image resolution or wide distance between adjacent image layers
- Reduction of number of tests–a desire to minimize the number of imaging sessions needed for a diagnosis.

Unfortunately, in the domain of general diagnosis the above guidelines are interpreted as trade-offs and the resulting models have either high performance but low transparency or high transparency but low performance. For example, the class of DSTs that use artificial neural network in general have good classification performance, but poor knowledge transparency when trained on a sufficiently large data sets. In contrast, Assistant-R, R-QMR, ISABEL and other DSTs are top down induction engines that use decision trees and expert knowledge ontology with high transparency but the performance is often not as high (Cestnik et al., 1987; Miller, 2016; Martinez-Franco et al., 2018). Due to the high stake consequences of making medical diagnostic decisions it is important that the proposed DST solutions meet all proposed guidelines.

The quality of the machine learning algorithms for simple tasks such as medical image segmentation or classification is continuously improving. That said, few diagnostic synthesis tools exits and even fewer are actually used by the radiologist (Shattuck and Leahy, 2002; Wang et al., 2016). The adoption criteria for the bio-medical imaging tools should include (1) what is the tool's knowledge transparency and the explanation ability, (2) low type I and type II error, (3) how much new, previously unavailable information the tool discovered and presented to a physician and (4) how much integration overhead did the tool introduce or how well does the tool integrate into the exiting image processing stream.

Unlike many machine learning applications, the use of computer vision algorithms as the diagnostic tools for processing brain scan images have direct impact on the patient life and well being. The type II errors of falsely inferring the absence of anomaly in the brain tissue is unacceptable, while a low false positive error (type I error) is allowed as the algorithm's purpose is to aid the diagnostic process and not to replace the human radiologist. The desired efficacy of the DSTs is to have high sensitivity and specificity scores to make the error repeated on multiple trials on a single patient highly improbable – a performance comparable to a human. Currently, the segmentation of brain soft tissue using machine learning algorithms simply does not have high enough sensitivity and specificity scores to be accepted as a medical support tool (Menze et al., 2015). Although generally DSTs still lag behind physicians, Menze et al. compared the performance of a radiologist against the latest machine learning algorithms on the benchmark brain tumor image segmentation images to conclude that the error between the human and the machine for the brain tumor segmentation are comparable (Menze et al., 2015). The expected sensitivity and specificity threshold for the processing of the brain scan images is still to be determined, but the application of the bio-medical diagnostic tools in other fields of medicine commonly exceed 95% sensitivity and specificity scores, and we expect a similar or higher threshold to be applicable for any clinically relevant DST.

The diagnostic process of a single case already requires several hours by a highly trained and specialized radiologist, so an introduction of a new tool has an associated cost-benefit trade-off. If the balance between the increase of the radiologist's decision making complexity exceeds the amount of the new information presented to a radiologist, the new tool hinders the diagnostic process and could lower the physician's diagnostic accuracy. For example, Kononenko's empirical studies showed that the physicians prefer the diagnosis and explanations by Bayesian classifiers and decision tree classifiers, such as the Assistant-R and LFC, due to their high knowledge transparency (Kononenko, 2001). In contrast, the top performing ML algorithms analyzing problems such as the brain tumor segmentation in the images (BraTS) or even a much easier task of recognizing and localize ordinary objects in the natural scenes using artificial neural network based algorithms have poor knowledge transparency and explanation ability (Kononenko, 2001; Krizhevsky et al., 2012; Menze et al., 2016).

The goal of AI based tools that support radiologist's analysis of brain soft tissue images is to reduce the diagnostic complexity and error while providing additional analytic insight with high knowledge transparency and explanation. The diagnostic accuracy using the ML data analysis has to be verified and compliant with the radiologist's knowledge and practice. The knowledge structure extracted from the images has to be supported by clearly identified lower level features that consist of the image artifacts already used by the radiologist, clearly identified artifacts that were not used to synthesize the extracted knowledge, or the new features not seen by the radiologists. The relationships between the features define the structure of the knowledge that can be argued by the radiologist in support of the final diagnosis. How the model learned to select the relevant features and the relationships among them does not have to mimic how the radiologists learn to diagnose a pathology as the AI's goal is to learn the complex features and interactions missed in the first place. That said, the newly discovered knowledge must be verifiable by the radiologist.

Imaging tests are ordered for a variety of underlying clinical complaints^1^to generate a differential diagnosis or to determine the extent or localization of a known pathology. Radiologists analyse the resulting scans with little knowledge of the patient's case other than some basic history, which helps prevent diagnostic bias. For the same reasons, we will survey only the methodologies that analyse brain scans without and do not use additional patient information as the algorithm's inputs. To further limit the scope, we will discuss the diagnosis of brain tumors and traumatic brain injury (TBI) as two brain pathologies representative of different diagnostic mechanisms.

## 3. Human performance

Extensively trained physicians remain the gold standard in information extraction and diagnostic synthesis from the radiology studies. Radiologists identify both discrete and diffuse features from the soft-tissue images and differentiate pathologies from imaging errors or other benign structural anomalies such as calcium deposits (Figure 1). Detecting an anomaly pattern in the soft tissue images of the brain is nonetheless challenging even for a trained professional, so specialized imaging modalities and image post-processing techniques are developed to aid the diagnostic process. The additional image sets and the output measurements from different imaging techniques contrast different aspects of the brain's physiology such as tissue density, fluid flow, volumetric measurements and electromagnetic properties. The radiologists extract information and cross-reference multiple sets of images for each patient to create a holistic picture of the information extracted from the large volume of raw image data. The final diagnosis is then inferred from the collection of the imaging findings.

Although radiologists are highly trained and have access to advanced tools to aid diagnosis, studies have found that two radiologists will disagree on a diagnosis between 5 and 20 percent of the time (Berlin, 2001). The opportunity for ML based decision support tools are to reduce diagnostic error by differentiating the competing diagnoses using newly discovered knowledge or presenting a ranked list of alternative diagnosis for further inspection, allowing the clinician to eliminate some diagnoses using additional, non-imaging based information about the patient's case.

Traumatic brain injury and tumor segmentation are two diagnoses that use brain scan images to diagnose pathology, monitor disease progression or recovery, or localize the damaged tissue for surgical intervention. More importantly, the selected pathologies represent two very different mechanisms of diagnosis that are representative of many brain imaging based diagnostic processes. Brain tumors are well defined regions in the brain that are identified from the images by the intensity indicating the tissue density and fluid content difference, the location is constrained to the main volume of the brain, and the smooth, 'globular' shape of the tissue. In other words, a discrete well defined feature(s) with well defined boundaries. Traumatic brain injury on the other hand, does not have a single tell-tale feature that identifies the pathology. A radiologist diagnoses this complex injury from a collection of partial anomalies. It is worth noting that any one of the anomalies occurring independently might not be a pathology, but instead may be a natural variation of the individual's brain structures. The third type of information extracted from the brain scans are diffuse features. For example, inflammation of brain tissue is localized, but does not have clearly defined boundaries like a tumor would. In brain scans, inflammation is identified as a region of higher intensity than the surrounding tissue. Although diffuse brain imaging features can be categorized on their own, there are no automated diagnostic tools yet. We will discuss them loosely in both contexts of tumor segmentation and traumatic brain injury.

Tumor detection is a common radiology analysis task that is diagnosed even in scans ordered for a tumor unrelated diagnosis. Tumor diagnosis can be confirmed from imaging tests using Computed Tomography (CT), Positron Emission Tomography (PET), or Magnetic Resonance Imaging (MRI). As radiotherapy and surgery are the most effective treatments to remove brain tumors, the accurate localization of the diseased tissue is critical. If all cancer cells are not removed the tumor can relapse. If normal tissue in addition to the tumor is removed, the brain's normal functionality can be disrupted. In addition to guiding surgical interventions, brain imaging is used to monitor a tumor's response to the treatment. A tumor can have four imaging features: edema (swelling near the tumor), contrast enhancement (related to “leaky” blood vessels and tumoral angiogenesis), necrotic tissue, and non-enhancing tumor. Accurate identification of each tumor tissue type is necessary as not all tumor affected tissue has to be removed or targeted for radiotherapy. Physician segmentation of tumor regions has significant variability between practitioners with the Dice score of 74–85% to accurately identify tumor vs. healthy tissue (Njeh, 2008; Menze et al., 2015).

Traumatic brain injury (TBI) is a global public health problem with 57 million annual hospitalizations with one or more TBI incidents per patient of which over 10 million result in death (Langlois et al., 2006). TBI can result from a moderate-to-severe head injury or long-term, repetitive, low-force impacts. TBI is common among athletes participating in contact sports, armed forces combat veterans, or general head trauma from accidents. TBI intensity is classified as mild, moderate or severe, and each requires different treatment plan. With decreasing severity of TBI injury, TBI becomes increasingly difficult to diagnose by a radiologist using brain imaging (Ghajar, 2000). Rapid TBI diagnosis is desired to ensure the patient's recovery and prevent further damage and consequent TBI progression. Currently, mild TBI is diagnosed from clinical evaluation using the Glasgow Coma Score (GCS) and symptoms involving the injury as no consistent, distinct imaging features are detectable by a radiologist to make the diagnosis (Williams et al., 1990). Although the sensitivity of the current imaging instrumentation is insufficient to definitively diagnose mild TBI, a combination of post-processing image processing algorithms, advanced imaging techniques, and improvement of the imaging resolution will be used in the future to aid in the diagnosis of mild TBI. The vast majority of patients with mild TBI make a full neurological recovery, however accurate diagnosis allows patients to recover more quickly and to avoid the risk of a second TBI event while they are recovering. Moderate TBI cases can be diagnosed from brain imaging by a radiology specialist trained in TBI diagnosis. In moderate TBI cases, the brain structures are altered from patient's normal which is difficult to quantify as patients normally do not have pre-injury brain scan of their healthy brains. The radiologist has to differentiate the injury from the expected variance to the brain structures. Patients with severe TBI have MRI scans with clearly visible changes to the brain structures; these patients are frequently comatose and unable to follow simple instructions (Ghajar, 2000). Severe TBI is generally diagnosable by any radiologist on MRI.

To formulate a TBI diagnosis, a radiologist cross-references multiple image sets to extract structural brain anomalies from normal expected variance. There are changes to the brain tissue which are visible on Diffuse Tensor Imaging (DTI), a technique that reveals the location, orientation, and directional anisotropy of water molecules within the brain. The resulting changes to the brain are visible as the asymmetric pattern of the brain's fluid movement and the loss of the brain's volume due to damage to the underlying white matter. As the brain volume decreases at the injury site, there is an increase in the amount of cerebrospinal fluid to fill this space.

## 4. Soft tissue imaging techniques

Several different techniques can be used for imaging of brain's soft tissue: radiography, magnetic resonance imaging (MRI), nuclear medicine, ultrasound, and infrared imaging. MRIs are most often used to train ML algorithms for brain anomaly identification. This is partially because the MRI imaging is used by physicians who provided the image annotations needed for model construction using supervised machine learning algorithms. Most available data-sets are annotated MRI images. Although MRI is the most common diagnostic imaging tool, we will briefly describe other imaging techniques for the completeness and the transference of image analysis methods between different imaging modalities. In other words, the AI and ML algorithms developed to identify structures and anomalies in one image type, can be used to analyse other image types by simply swapping the image sets.

Radiography uses a wide x-ray beam for imaging both bone and soft tissue. Radiography is widely used due to its low cost and high resolution of resulting images. The use of x-ray has its drawbacks: it is harmful in the case of frequent or high intensity exposures, imaging sensitive tissues, and for at risk patients such as pregnant women. Radiography is also limited due to its planar acquisition technique with overlapping structures projecting onto a single image. This severely limits evaluation of underlying structures with visualization of individual organs, especially brain, made impossible by the overlying dense bony calvarium.

Nuclear medical imaging is an effective technique to assess the structure and function of an organ, tissue, bone, or the entire system. This technique uses chemical contrast agents injected into the body that react with proteins or sugars to be recorded by the imaging detectors. The required use of radionuclide markers have to meet strict guidelines to be safe for repeated application. Although nuclear medical imaging can visualize a limited range of brain functions, it produces relatively low resolution images. These factors limit its use to localize active sections of the brain during or immediately after seizures for surgical intervention, to diagnose early onset of Alzheimer's or Parkinson's disease, or for brain tumor activity in the assessment of radiation necrosis vs. an actively growing tumor.

Ultrasound is an imaging technique used to examine brain tissue and the blood flow to the brain. As a non-invasive procedure, it is effective in diagnosing a relatively wide range of conditions including the narrowing or enlarging of intracranial vessels, congenital brain anomalies, monitoring the dynamics of cerebral fluid, assessment of damage to the brain's white matter, tumor site localization, intracerebral hemorrhage, assessment of stroke risk in adults and vascular disease related to sickle cell disease. Although the ultrasound based imaging techniques do not produce high resolution images in comparison to conventional x-ray or MRI, dynamic cine images and Doppler flow measurements can produce real-time visualization of blood and cerebrospinal fluid dynamics. In addition to brain tissue imaging, ultrasound is commonly used for imaging other organs such as the heart and lung.

**Figure 2:**
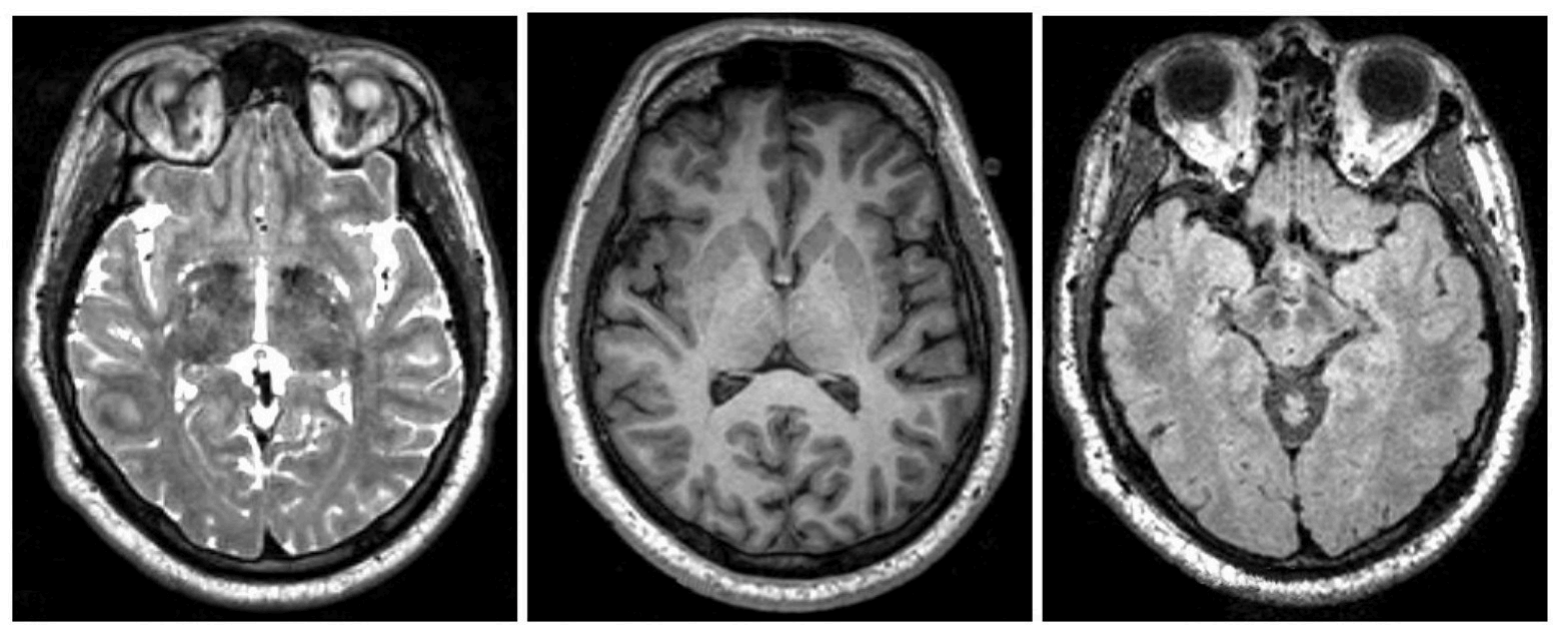
Examples of different MRI imaging techniques. Left: T2 image. Middle: T1 image. Right: FLAIR image. Table shows the brightness of the tissue using the three most common MRI imaging technique from dark to bright (Preston, 2006).

Magnetic Resonance Imaging (MRI) is a highly available, non-invasive imaging technique that uses powerful magnets to make detailed tomographic images (Figure 2). Since there is no known negative long-term effect from exposure to strong magnetic fields, a physician can use repeated testing to monitor brain tissue recovery or narrow a differential diagnosis. MRI scanning is limited in patients with implanted medical devices or those patients with metallic fragments which are ferromagnetic and in dangerous locations. MR imaging equipment cost is relatively high, but one might argue that the limiting factor in the MRI application is the lack of radiology specialists to analyse the images and synthesize the information to form a diagnosis. MRI has several specific sequences utilized which are discussed in detail in the next section.

The image analysis of brain's soft tissue can be categorized into three scopes: local, regional, and global. The local features visible in the brain MRI images can range from a few millimeters to several centimeters. A feature to be classified as an anomaly is either an unexpected mass that does not belong at the given location or a significant variation of the expected brain structure from the normal shape and size.

Discrete anomalies localized with well-defined edges include tumors, foreign bodies, hemorrhage, and swelling. Tissue infection or inflammation on the other hand are usually more diffuse anomalies in the imaged brain tissue, only visible on the regional or global scale.

Detection of regional anomalies requires identification of structural shape asymmetry, signal intensity variation between local brain structures and the surrounding tissue, color asymmetry variation that identifies the difference in the fluid flows, or unexpected volumetric differences in the tissue. The global scale analysis provides the context for the features detected on the local and regional levels to decide if they are indicative of a pathology or an expected variation.

The global analysis by a radiologist is challenging as a single local scope feature may be classified as a minor difference in healthy tissue but the presentation of several such features as a group could indicate an illness. The detection and classification of discrete, structural brain anomalies can be easily recognized by an appropriately trained health professional, especially if the anomaly is large and a well-defined discrete mass. However, a diagnosis of a moderate TBI that is manifested by regional or global relationships between features is only possible by highly-trained specialists with years of experience. Additionally, the in-depth knowledge of different imaging techniques is necessary to cross-reference to verify the consistency of the candidate features across multiple image sets. The ability to reason about the detected features in multiple image sets on different scope achieves the desired transparency in formulation of the medical diagnosis and the course of treatment.

Radiologists commonly diagnose patient's brain pathology using MRI based imaging that mostly replaced computed tomography (CT) as the imaging technique of choice. Image analysis is not only used for the initial diagnosis, but also for the preoperative staging, assessing the effectiveness of the treatment plan and other aspects of medical care (Berquist et al., 1990). MRI image processing offers visualization of the brain anatomy in multiple planes (axial, sagittal, coronal, oblique, and others depending on the gradient coils orientation) and reveals details about the brain's (static) structures as well as information about fluid movement and underlying tissue integrity (Preston, 2006).

### 4.1. MRI imaging and processing techniques (T1, T2, FLAIR, and DTI)

MRIs of the brain produce two-dimensional views of the soft tissue, where each image represents one horizontal cross-section (axial anatomical plane slice) with two consecutive images separated from each other along the vertical axis (spinal axis) between 0.2 and 6.0*mm*. Based on the distance between the vertical slices, the resulting image sets have between 170 and 1, 500 images depending on how much of the brain imaging is desired and how many different imaging instruments were used.

Although the imaging machine can be configured and focused using multiple anatomical templates or Brodmann Maps, the common MRI imaging for the initial diagnostic image the entire brain cross-section with 1*mm* spacing between vertical slices (Tzourio-Mazoyer et al., 2002). BTATS is a publicly available data set that was constructed with the above described configuration and is commonly used as a benchmark for the training and validation of the brain tumor segmentation algorithms (Menze et al., 2015, 2016).

### 4.2. MRI image pre-processing and baseline measurements

The artifacts introduced into the raw images by the imaging hardware used must be first removed before the diagnostic level algorithms are used to process the MRI sets. Many mature, open source algorithms exist to perform these low level tasks, but the demand for higher accuracy correction algorithms remains as the imaging resolution increases (see section 6.1 for details). The most common correction tasks include: topological correction, axial correction, non-uniformity correction, skull-stripping, and tissue classification. The healthy imaged tissue should be a closed contour or surface (after 3D reconstruction). The topological correction algorithms will repair any holes and protrusions introduced into the images during imaging or initial processing (Shattuck and Leahy, 2001). Many of the brain's functions are studied by a radiologist as a symmetric pattern between the brain's hemispheres. The multi-axial correlation will adjust the orientation of the 2D slices to be perpendicular to the vertical axis so the brain structures visible on each slice show the matching brain structures rather then the parts of the brain from a horizontally misaligned part of a brain. The intensity of the imaging beam used by the MRI device will attenuate when imaging tissue that is farther away from the beam's source. The intensity non-uniformity adjustment will remove the color gradient difference caused by the attenuation in the 2D slices as the raw images are lighter closer to the beam's source (Belaroussi et al., 2006; Mingsheng Chen, 2017). The low level MRI retrospective image algorithms can be grouped into grayscale level based and transform based categories and include additional corrections that include surface fitting, spatial filtering and image enhancements tasks (Belaroussi et al., 2006).

The skull stripping and tissue classification could be considered higher order image processing tasks depending on the desired classification and localization accuracy (Bakas et al., 2017a,b). Several simple algorithm successfully implement a crude version of the skull-stripping that will extract only the brain's soft tissue structures from the MRI images and mask off the surrounding tissues of the skin, bone, cartilage etc. (Hahn and Peitgen, 2000; Lee et al., 2003; Ségonne et al., 2004; Zhuang et al., 2006). The tissue type classification in the most simple form will differentiate the brain's white from gray matter, but advanced ML algorithms for tissue type classification can be used for the tumor localization or identification of de-myelinated tissue (Cocosco et al., 2003; Ferreira da Silva, 2007; Li et al., 2009; Basser and Pierpaoli, 2011).

The second class of the image analysis challenges is related to the high variation of human brain physiology visible in the healthy soft-tissue images. Studies of fMRIs of left-handed and right-handed individuals show that leftward asymmetry in language-related gray and white matter areas of the brain to be proposed as a structural correlate of left-sided functional hemispheric language lateralization, but that the neuroanatomical basis of the reported volumetric white matter asymmetry is not fully understood (Vernooij et al., 2007). Highley et al. studied the differences in the female and male fiber composition in the corpus callosum and found dis-proportionally higher fiber density in the female brains (Highley et al., 1999). The fiber density is only one of many tissue differences that are clearly visible in the MRI. Thus far, there is no automation in differentiating the normal variance of the brain's physiology from the anomalies that indicate certain illness, instead radiologist's expert knowledge is needed to distinguish the allowed tissue variations from pathological anomalies by integrating the knowledge extracted from different data-sets and the features identified anywhere in the MRI images.

### 4.3. MRI imaging techniques

Specialized MRI techniques were developed to image different structures and bio-mechanics of the brain. Figure 2 shows the most common MRI imaging techniques T1-weighted, T2-weighted, and fluid attenuated inversion recovery (FLAIR) images. All techniques use a radio frequency pulse and changes in the magnetic field that allow for measurement of phase and frequency changes within the tissue. Tissue with short T1-relaxation time appear brighter in a T1-weighted image, tissue with long T2 relaxation time appear brighter in a T2-weighted image, and a FLAIR image is similar to a T2-weighted image except that the cerebrospinal fluid (CSF) tissue is suppressed (Weishaupt et al., 2008). For example, the fatty tissue quickly returns its longitudinal magnetization, so it appears bright in T1-weighted images, but the cerebrospinal fluid on the other hand has less magnetization after being exposed to the gradient. The fluid will appear dark due to its low signal. Figure 2 shows brightness of the tissue with the differing imaging techniques.

MRI is particularly useful to reveal different characteristics of the specific tissue. Cross-referencing the multiple image sets created by T1, T2, and FLAIR MRI techniques, specific characteristics of a tissue structure will become apparent. For example, just by looking at the reference table in Figure 2, the areas of tissue with inflammation appear dark in a T1-weighted scan and bright in a T2-weighted scan.

The reference table is simplified to only 4 different tissue types and 3 different imaging techniques, whereas in practice, a radiologist can identify several 100 different tissue characteristics and using other imaging techniques to confirm or disqualify differential diagnosis. The identification of large structural changes such as a large tumor or a severe TBI can be diagnosed using T1, T2, and FLAIR MRI, but the heuristics to identify smaller injuries and tumors are more difficult to define. For example, the inability to diagnose mild TBI is because there is no single clearly defined feature that identifies the injury, instead it is a combination of several signs at different areas of the MRI images that determine a diagnosis. That said, Weishaupt et al.'s studies recommend that even the straight forward diagnosis of the large structural changes such as tumors can benefit from cross-referencing the image sets from all available imaging techniques to reveal all different tumor sub-components, surrounding inflammation, fluid accumulation etc., as no imaging technique alone shows all tumor aspects (Weishaupt et al., 2008). This is an opportunity to build intelligent machine learning algorithms in tandem with medical imaging for cross image-set integrative tissue type classification to augment human intelligence of tissue characterization.

**Figure 3:**
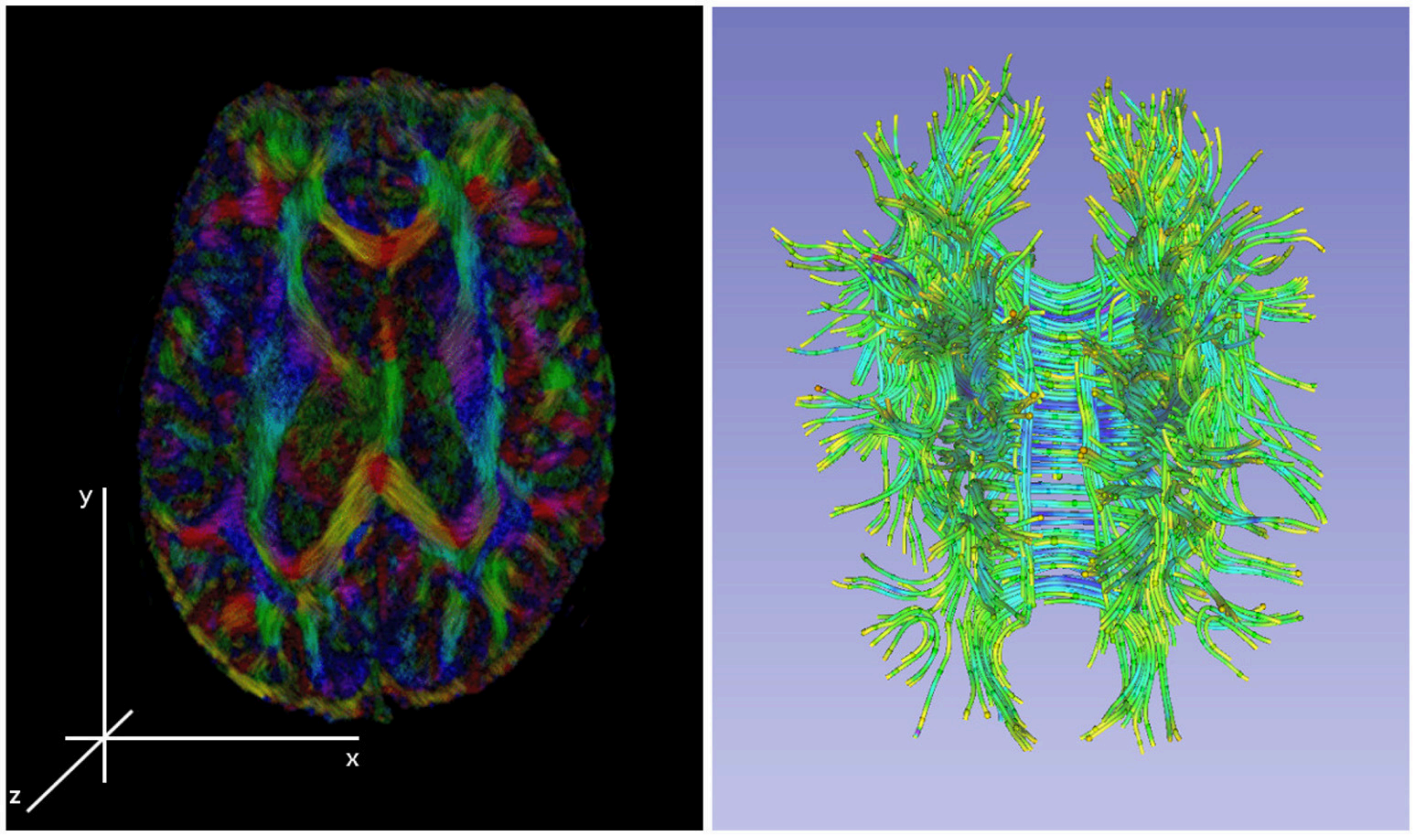
**Left**: an illustration of a diffusion tensor imaging 3D reconstruction. **Right**: the tractology image created by post-processing of the 3D DTI reconstruction from the DWI sources. The tractology image shows 3D view of individual fiber tracts and the color indicating the fluid flow direction within the tract.

Figure 3 shows Diffusion Tensor MRI (DT-MRI), a result of post-processing technique applied to the Diffusion Weighted Imaging (DWI) modality, as a MRI based imaging technique that allows for highly detailed images of soft tissue that allows for new way of examining the connectivity of the brain's tissue (Basser and Jones, 2002; Jones and Leemans, 2011; Soares et al., 2013). Unlike conventional anatomic MRI, diffusion tensor imaging (DTI) produces values of each voxel calculated from six or more diffusion measurements and from different orientations of the diffusion sensing gradients. DTI measures local diffusion characteristics of water molecule displacement averaged over a voxel. The resulting images show voxel level, multi-slice or three dimensional composites of the tissues and organs. Unlike other MR images, DT-MRI provides eigenvalues of the diffusion tensor, its trace measures of the degree of diffusion anisotropy and organization that can be used to estimate the fluid movement directions, flow volumes and the fiber densities (Basser and Jones, 2002). DTI's use in mapping the fibers' connectivity to reveal regions with restricted profusion or disconnectedness that is essential for stroke or moderate TBI diagnoses. Note that the information of the local flow dynamics is not available in the conventional MR images that produce a static, low-resolution snapshot of the tissue (Gonzalez et al., 1999).

DTI imaging provides significant opportunities for machine learning community as the DT-MRI based image sets contain additional information that is missing in T1, T2, or FLAIR MRI modalities. DTI images have much higher resolution needed for extracting the tissue features, the voxel-level raw measurements are unused by a physician and only used as an average (lossy) value for visualization, and the color information provides additional dimensions to extract the flow dynamics from the images. The nature of MRI as a 3D imaging technique has its benefit, namely the images are construction as 3D measurements which captures all information needed to build 3D views without requiring additional image post-processing. The resulting 3D composites have less imaging error that was introduced by the imaging robotics or the post-processing of the output images. That said, there are no studies that use 3D DTI composites for processing as the basis for medical diagnosis. This rises a questions about the trade-off of using the 3D composites that require additional computational resources and the ability to produce additional knowledge from the data needed for feature extraction and subsequent diagnosis. Havaei et al. compared the performance of a machine learning algorithm using the 3D MRI composites with the 2D image version and concluded that the algorithm that used 3D composite did not improve the localization accuracy for the tumor segmentation task, but performed slower then training on 2D images (Havaei et al., 2017).

Machine learning algorithms were successfully used to understand the correlation between the genetic micro-deletions and the cognitive brain functions from the DTI images (Tylee et al., 2017), decrease the MRI scan acquisition time by twelve-fold using deep neural networks (Golkov et al., 2016), extract discriminant features from DTI images to build a simple support vector machine (SVM) as well as more sophisticated classifiers to diagnose the Alzheimer disease (Haller et al., 2010; Graña et al., 2011; Beheshti et al., 2017a,b; Altaf et al., 2018), process the fiber tract-based spatial statistics to train a model to diagnose the Parkinson's disease (Haller et al., 2012).

The latest MRI modalities applied on tumor diagnosis include Dynamic Contrast Enhanced (DCE) imaging (Lin et al., 2017), Diffusion Kurtosis Imaging (DKI) (Alexander et al., 2017), Magnetic Resonance Spectroscopy (MRS) (Alexander et al., 2017), Chemical Exchange Saturation Transfer (CEST) (Van Zijl and Yadav, 2011) as well as the associated image processing derivatives Arterial Spin Labeling (ASL) (Telischak et al., 2015), Dynamic Susceptibility-Weighted Contrast (DSC) (Soni et al., 2017), Intravoxel Incoherent Motion (IVM) (Lin et al., 2015) to name a few. Please see Alexander et al. (2017) for a comprehensive review of the latest imaging modalities and their derivatives. To the best of our knowledge, the above methods were used in clinical settings and not by a machine learning algorithm to build tumor detection models. With time and clinical use, these techniques will produce datasets that will provide significant opportunities to automate the analysis of the brain's soft tissue. On contrary, De Visschere et al. observed that some of the new techniques might not increase the classification accuracy when used by a physician (De Visschere et al., 2017). Whether or not this observation will hold true in the future, when automated machine learning algorithms are used to analyse the image sets from the new imaging modalities, remains to be seen.

### 4.4. 2D analysis vs. 3D reconstructions

Due to limited application of ML using DTI images, basic studies are needed to understand how to build better ML algorithms using 3D DTI composites for diagnostic purposes that utilize the color information, encode the 3D topologies of the fiber structures, and correlate the features extracted from the DTI composites with the features extracted from other imaging techniques. For example, moderate TBI diagnosis is based on the DTI images as a lack of anisotropic diffusion at the primary injury site that is significantly different from the diffusion in the same region in the opposite brain hemisphere, AND the primary injury region has smaller volumetric measurements in the 3D MRI composites in comparison to the healthy tissue, AND there exists a brain region that has increased volume caused due to the displaced cerebral fluid from the primary injury site.

Although 3D brain reconstructions are currently available to the radiologists for both MRI and DTI image sets, radiologists examine and collate features primarily from 2D images. The 3D spatial distribution of the tissue features is crucial for accurate diagnosis and treatment planning. The radiologists do not use any additional tools to construct the 3D relationships among the detected features, so the integration is constructed as the radiologist's abstract or mental picture. Engineering of a ML algorithm as a higher order DST has to accurately encode these relationships by either (1) explicitly recording the 3D coordinates of the feature's location, orientation, shape properties extracted from multiple 2D slices as the features commonly span more then one slice or (2) use the 3D composite that retains all these quantities, but is computationally much more expensive to process. Creating the 3D MRI composite is done by stacking of the 2D pre-processed slices using registration points on each slice for the mutual slice alignments. The 3D reconstruction is possible for many of the imaging modalities such as tomography and functional magnetic resonance (T1, T2, FLAIR, etc.) or fiber-tracking derivative of the DTI. Very few MRI or non-MRI, open data sets are available for the 3D reconstruction, and even fewer have radiologist's reads with associated labels (annotations) that are needed to train and evaluate a ML model.

The ML algorithms that use the 3D composites to explicitly represent the MRI scan for processing have to be supported by massively parallel hardware architecture. The feature extraction requires large portions of the 3D data-structures to be memory resident for the algorithm to extract the tissue features at spatially explicit local and relate these to the detected features at other locations. To address this challenge, alternative data-structures are needed for efficient and lossless data representation along with the algorithms for fast manipulation of the data-structures. For example the fiber 3D reconstruction from DTI images is only concerned with the location, volume and flow information of the fibers with the rest of the imaged brain volume being irrelevant and does not have to be represented in the 3D data-structure. This alternative DTI projection is called tractography and even though it only shows the fluid carrying in the fiber tracks the data-structures used to represent the 3D composite is in a 3D pixel space, or “voxel.” An alternative vector space or as continuous fields in geo-spatial computation could be used to represent only the regions on interest (Mostafavi et al., 2010; Beni et al., 2011).

The alternative approach is to use 3D images to segment a region of interest first then perform the object classification on a much smaller 3D voxel structure which will decreases the hardware requirements needed and the computing time. Multiple studies showed that the results accuracy increases if the region of interest (ROI) is extracted first with the subsequent object classification (El-Dahshan et al., 2014; Shankar et al., 2016; Yu and Yang, 2017).

Finally, 3D composites can be used for clinical diagnosis only if the software is Food and Drug Administration (FDA) approved. A majority of the mature, well supported, open-source software packages (such as 3D Slicer^2^) are not FDA approved and are mainly used for biomedical academic research (Fedorov et al., 2012).

## 5. The AI and machine learning for brain pathology diagnosis

Machine learning is a field of computer science that builds mathematical models to recognize patterns in data to help humans make better decisions (Alpaydin, 2014). Computer vision is a sub-field of machine learning that trains computers models to extract and analyse information from images (Shirai, 2012). Several computer vision algorithms applied on the object recognition problems achieved classification accuracy results better than the best human performance (Krizhevsky et al., 2012; Russakovsky et al., 2015). Despite the success of these algorithms on a narrow problem domain of object detection and localization tasks, analyzing images for content analysis regardless of the domain remains to be difficult and open problem (Pal and Pal, 1993; Xiao et al., 2010). The application of machine learning and computer vision algorithms to analyse brain scan images is mostly unexplored (with exception of the brain tumor segmentation challenge; Menze et al., 2016,^3^).

The training and evaluating of computer vision models often relies on very large data-sets, clearly and accurately annotated ground-truths in the images, and a powerful hardware architecture to support the computational requirements of the proposed algorithms. The task of scene and object recognition is similar for the scene image processing and the MRI image analysis. In particular they both contain many object (scene) or structure (MRI) classes, the information in the images is unstructured with a significant amount of noise, the models have to extract, analyse, and synthesize features on multiple scopes.

That said, the transference of the existing methodologies to the MRI analysis has several challenges. The best performing computer vision algorithms on the multi-class scene recognition task had an average accuracy of 81.1−100% when trained on 1.3−80.0 million images. The model complexity has to be sufficient to generalize and extract the features from a large data sets which results in the models with millions of parameters and thousands of logic units (Torralba et al., 2008; Krizhevsky et al., 2012). Finally model construction and validation has to be supported by a massively parallel hardware architecture. The analysis of MRI images is unlikely to use data collected from more than several 100 patients, the models have to be equally efficient and accurate in extracting features and relationships and provide a new knowledge to a radiologist, and the hardware has to support the processing of the high resolution 3D image composites.

One way of addressing these challenges is not to rely on the computer vision algorithms to fully automate the feature extraction from the images, instead use the radiologist's process based heuristics and engineer the low level feature extractors, measure the difference of the detected structures from their expected size, compute the reflection similarity of the corresponding brain centers, and build custom hardware to support the computation. The methodology of building the radiologist's decision support tools should trade the the deep, automatic models for hierarchically constructed ensemble of models with high knowledge transparency.

**Figure 4:**
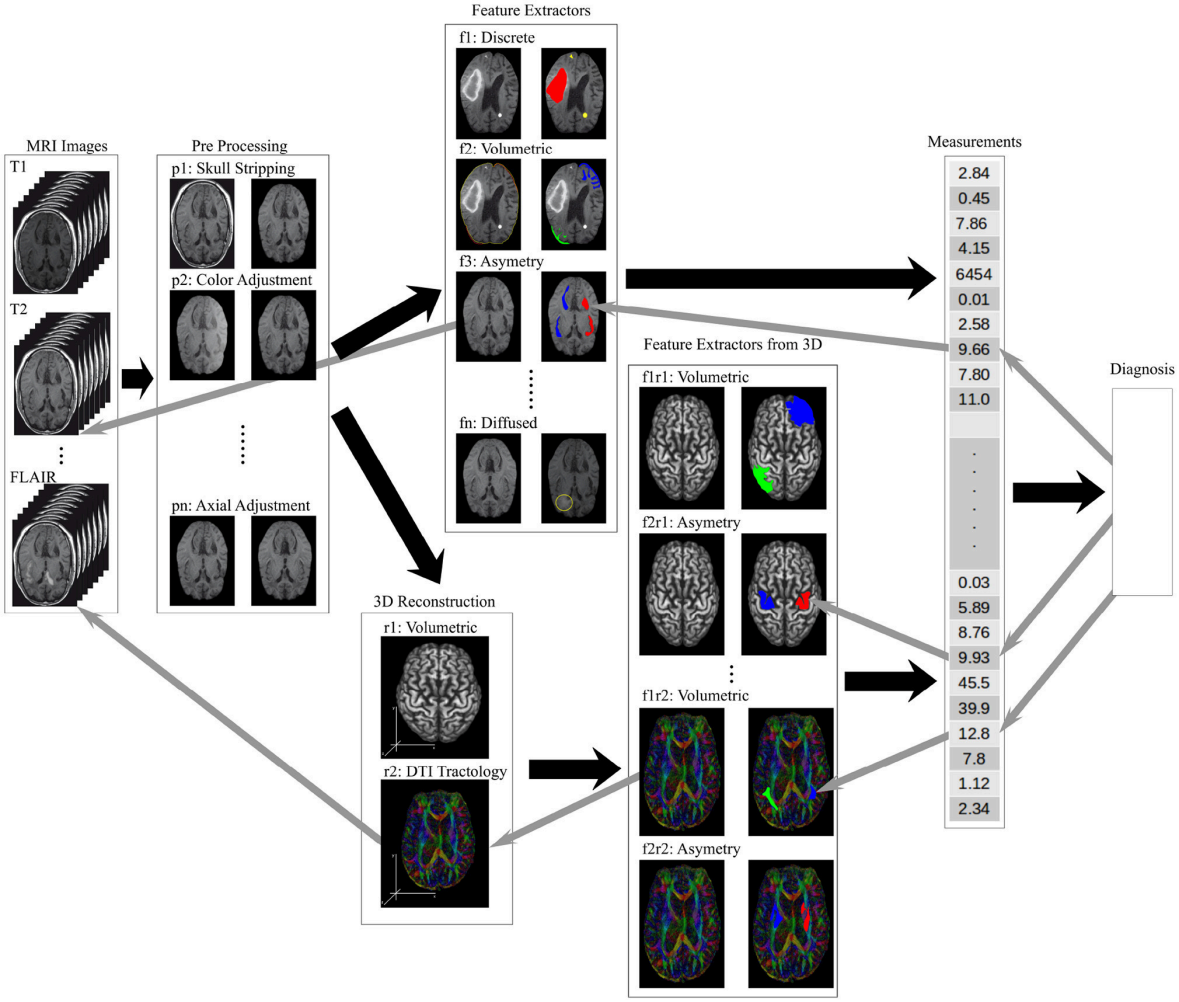
An illustration of the information flow through a data processing framework needed to analyse brain MRI scans to produce a diagnosis. The raw image sets are first pre-processed (tasks labeled **p-**), the structural features are extracted (tasks labeled **f-**), higher order features and measurements calculated from 2D and 3D composites (tasks labeled **r-** and **f-r-** respectively), and the final diagnosis model synthesizing the measurement vector of local, regional and global features. The back arrows show the flow of information while the gray arrows show the trace-back feedback needed for a transparent decision support tool used by a radiologist.

The hierarchical stacked models implement the work flow needed to process raw images and produce actionable knowledge for a radiologist. Figure 4 shows a hierarchical organization of models where each image processing task is implemented as a standalone model. The right arrows show the processing stages of the image analysis and information extraction. The left arrow show the knowledge transparency for a radiologist to back track the heuristics used by the model ensemble to form the final diagnosis.

Figure 4 shows the pre-processing models, described in the previous section as tasks **p-**, to regularize the images by resizing, rotating, and removing the unwanted artifacts from the image. The soft-tissue of the brain is extracted as a region of interest (ROI) by removing all other structures from the images (Bakas et al., 2017a,b). The low level feature extraction models, labeled **f-**, generate a feature space used for the structural measurements and the learning process. The higher order features are extracted from the 3D composites or measured from the 2D images.

The ability to identify and compare the higher order features requires registration of brain structures – a task where the anatomical regions of the brain are detected and labeled which allows for a measurement of structural similarities across brain structures. The similarity measures use a variety of comparative morphometry and group analysis algorithms (Klein et al., 2009). An accurate registration allows for the anatomical region segmentation and subsequent comparison of the region's volume against the expected (Greitz et al., 1991; Ardekani et al., 2005). The volumetric analysis of the MRI based regional segmentation was successfully used to diagnose Autism (McAlonan et al., 2004). Finally, the visualization and 3D reconstruction are not feature extractor models, non-less they are used in parallel with the feature extractor models to verify the model's results.

The last prediction in the hierarchically stacked models is the synthesis of the diagnosis that uses the input vector of the local, regional, and global feature measurements to predicts the diagnosis. This high level reasoning model can be implemented using many different methods including probabilistic models (Szolovits and Pauker, 1978), decision-theoretic expert systems (Horvitz et al., 1988), fuzzy set theory (Adlassnig, 1986), neural networks (Amato et al., 2013) and ontological based reasoning (García-Crespo et al., 2010; Bertaud-Gounot et al., 2012). The essential quality of these high level reasoning engines is its transparency and the ability to trace back the diagnostic decision through the ensemble of models used to support the diagnosis. Figure 4 shows the knowledge transparency as the gray arrows that identify the key measurements, that are traced to the high level features and weights, supported by the structures in the 3D composites, that lead all the way to the low level features and image artifacts.

It is worth noting that the image processing work-flow should be flexible and configurable to select the alternate processing models to diagnose different pathologies. For example the pre-processing may or many not include image normalization, orientation, or image resizing. Feature extraction can be minimal using the pixel intensity values alone to create the feature set, or use complex spatial statistics to define the features of interest, or a combination of both. The diagnosis prediction should be agnostic of the input vector size or its composition. Creation of a transparent machine learning model aims to help practitioners find the image patterns that go unnoticed, and become a tool for an early stage disease diagnosis, a treatment monitoring, an analysis of brain's structures, tissue or brain center segmentation, and diagnostic prediction.

### 5.1. Model and diagnostic performance

The goal of the AI and ML based applications in the medical community is to build medical decision support tools for reducing the complexity of the practitioner's work, not replacing it. Current benchmarks for machine learning applications generally look to achieve results similar to inter-practitioner error rates (Krizhevsky et al., 2012; Menze et al., 2016), but achieving these standards does not necessarily mean that the ML architecture will be usable in the medical practice (Menze et al., 2016). Although no qualitative research is available to explain why medical practitioners fail do adopt these tools, Kononeko et al. argue that the perceived cost of adding new tool and the increase in the decision making complexity for a diagnostician is likely a significant contributor to slow adoption (Kononenko, 2001). However, he also suggests that the usage of AI and ML based tools in the medical field is inevitable as the diagnostic accuracy is continuously improving. Menze et al.'s bibliometric analysis supports this proposition with the observation that the number of publications in the field of automated brain tumor segmentation has grown exponentially in the last few decades (Menze et al., 2015). Results in both fields of the automated brain tumor segmentation (BraTS challenge) (Menze et al., 2015, 2016) and the more general scene image analysis show continuous improvement (Krizhevsky et al., 2012) which attests to the interest in the problem, broader societal impact and the resulting financial support and the synthesis of new findings.

The renascence of machine learning and computer vision in recent years has produced a number of new brain MRI processing architectures (Menze et al., 2015, 2016; Havaei et al., 2017). The highest accuracy results for the medical image segmentation and image classification were achieved using Neural Networks. Convolution Neural Networks (CNN), a type of neural network, are especially popular and achieve high classification accuracy due to the algorithm's ability to learn complex hierarchy of features from generic inputs (Havaei et al., 2017). CNNs have been applied for segmentation of brain tumors in the BRATS challenge dataset and for microscopic cell segmentation (Ronneberger et al., 2015; Havaei et al., 2017). Top results on the BRATS challenge dataset, ImageNet dataset, and the Keggle Data Science Bowl all use a form of CNN architecture.

There are currently two main applications of ML on soft tissue images: soft tissue segmentation and diagnosis classification (El-Dahshan et al., 2014; Menze et al., 2016; Shankar et al., 2016; Alakwaa et al., 2017; Havaei et al., 2017). Tissue segmentation requires the AI implementation to identify and correctly label the different types of tissue within an image. Diagnosis classification requires the AI implementation to classify which diagnosis a particular image would be associated with (e.g., cancer or no cancer, tumor or no tumor). Both problems have found the usage of multiple soft-tissue imaging techniques as input to the ML to provide increased accuracy results (El-Dahshan et al., 2014; Menze et al., 2016; Alakwaa et al., 2017).

The complexity of medical image analysis can be illustrated on the segmentation task using MRI is the BraTS research (Menze et al., 2015). The goal of the BRATS research is to correctly identify the active tumor regions and its extensions within the brain and label the tumor's different regions accordingly (Havaei et al., 2017). A healthy brain has 3 types of tissues: white matter, gray matter, and the cerebrospinal fluid. Using several different image sets of the brain: T1 (spin-lattice relaxation), T2 (spin-spin relaxation), diffusion MRI, and fluid attenuation inversion recovery (FLAIR) pulse sequences. Segmenting the tumorous regions in the brain involve finding four distinct tumor regions: edema (swelling near the tumor), enhanced tumor (active tumorous tissue), necrotic tissue, and non-enhanced tumor. Accuracy on segmenting complete (all four tumor structures), core (all tumor structures except edema), and enhancing (region of only the enhanced tumor) is calculated using dice, sensitivity, and specificity (Menze et al., 2010) as follows:

(1) $$\begin{array}{r} Dice\left(P,T\right)=\frac{\left|P_{1}\wedge T_{1}\right|}{\left(\left|P_{1}|+|T_{1}\right|\right)/2} \end{array}$$

(2) $$\begin{array}{r} Sensitivity\left(P,T\right)=\frac{\left|P_{1}\wedge T_{1}\right|}{\left|T_{1}\right|} \end{array}$$

(3) $$\begin{array}{r} Specificity\left(P,T\right)=\frac{\left|P_{0}\wedge T_{0}\right|}{\left|T_{0}\right|} \end{array}$$

where P is the model prediction, T are the ground truth labels, and *T*_1_ and *T*_0_ are the positives and negatives for the tumor region in question. Similarly for *P*_1_ and *P*_0_. There is considerable disagreement between inter-practitioner segmentation (Dice scores in the range 74−85%) of low grade and high grade tumors (Menze et al., 2015). Notably Menze et al. shows that Dice score distribution is quite high with standard deviation of 10% and more with the most difficult tasks (tumor core in low-grade patients, active core in high-grade patients) (Menze et al., 2015). Multiple algorithms were tested on the BraTS data set, but no single method performed best for all tumor regions considered. However, the errors of the best algorithms for each individual region fell within human inter-rater variability (Menze et al., 2015).

The images from the BraTS data set contain only four soft tissue imaging techniques: T1, T1-weighted, T2-weighted, and FLAIR. The diffusion tensor MRI is not available for the BraTS data set and therefore it is not known how effective the ML algorithms are using this additional imaging modality on the tumor segmentation. The BRATS data set is pre-processed to the same anatomical template (axial orientation), interpolated to the same resolution (1*mm*^3^), and skull stripped (removal of any non brain tissue structures such as the skull, eyes, jaw etc.) (Menze et al., 2015; Bakas et al., 2017b). The annotation of the training and validation sets was done and approved by experienced neuro-radiologists. The annotation labels identify the four tumor substructures of (1) edema, (2) non-enhancing solid core, (3) necrotic or fluid-filled core, and (4) non-enhancing core. Menze et al. propose subsequent evaluation ranking that is based on Menze et al. (2015):

1. the “whole” tumor region including all four tumor substructures
2. the tumor “core” region including all tumor structures except the edema
3. the “active” tumor region only containing the enhancing core structures that are unique to high-grade cases

Many of the top ranking algorithms on the 2013 BraTS challenge produced their own low-level features as a first step and applied a discriminate classifier in the second step, transforming local features into class probabilities (Menze et al., 2015). The discriminant classifier algorithm was implemented using a random forest approach. On the 2016 BraTS challenge the top performing algorithms used a CNN for its segmentation process. In 2017, the research methodology proposed (Havaei et al., 2017) implementing a CNN on the 2013 BraTS data set to learn the low level features and produced the highest DICE scores. Accuracy continues to improve and the 2017 BraTS challenge included an additional challenge to predict patient survival associated with each image (Bakas et al., 2017b)^4^.

A variety of algorithms can be used as a discriminant classifier making the final medical diagnosis. To illustrate a broader range of the medical diagnosis algorithms, we will temporarily expand the discussion's scope to include the not only the brain tumor but also the lung cancer classifications. For brain tumor segmentation El-dashan et al. were able to produce the classification accuracy of 99% using a Feedback Pulse-Coupled Neural Network (FPCNN), a type of CNN architecture, for ROI segmentation and a feed forward ANN for image classification (El-Dahshan et al., 2014). Shankar et al. achieved an accuracy of 95.67% using the Gustafson-Kessel fuzzy clustering algorithm for classification (Shankar et al., 2016; Yu and Yang, 2017). Alakwaa et al.'s implementation used a CNN inspired architecture called U-Net to extract the ROI and the subsequent 3D CNN to classify the abnormal vs. normal tissue in the lung images (Ronneberger et al., 2015). Their resulting classification accuracy on the test set was 86.6% (Alakwaa et al., 2017). Alakwaa et al.'s CNN architecture used the 3D ROI composite of the lung which favors the use of 3D composites over the 2D image slices to extract the features and the relationships among the features. Alakwaa et al.'s implementation outperformed all available computer-aided diagnosis systems (Alakwaa et al., 2017). Classification accuracy generally uses the statistical measures of a binary classification test [true positive (TP), false positive (FP), true negative (TN), false negative (FP)]. The resulting classification accuracy is calculated using:

(4) $$\begin{array}{r} Accuracy=\frac{TP+TN}{TP+TN+FP+FN} \end{array}$$

### 5.2. Convolution neural networks

A convolution neural network (CNN) is an artificial neural network inspired architecture that extracts image features with increasing complexity or structure by alternating the convolution and pooling filters on the input implemented as respective neural network layers. The convolution filers can be randomly sampled from the inputs (imprinted), engineered by a human, or set at random and pruned by reinforcement during the model training. The output of the CNN architecture is either the final vector of detected features or the architecture will include the processing of the feature vector and produce the final classification through a fully connected layer (Fukushima and Miyake, 1982; Poggio and Girosi, 1990; Lo et al., 1995).

ML architectures for image classification have improved significantly following the work of Krizhevsky et al. on the ImageNet visual recognition database in 2012 using a CNN (Krizhevsky et al., 2012). In 2015, the best results on the ImageNet database were achieved using the CNN and to this day no other type of architecture produced better scene classification results (Russakovsky et al., 2015; Havaei et al., 2017; Hornak, 2017). This improvement to ML architectures translated to the image segmentation and classification for the medical image processing. In 2016, half of the submitted solutions for the BraTS image segmentation problem used a CNN as a feature extractor sub-model within the overall ML architecture, and many of these were among the top performing algorithm implementations (Menze et al., 2016; Havaei et al., 2017). Other medical image classification problems such as lung cancer classification and brain tumor classification also commonly use the CNN as a feature extractor from 2D images and 3D composites (El-Dahshan et al., 2014; Shankar et al., 2016).

Many recent publications report the variations of the baseline CNN and decoupling the feature detector (CNN) from the classification models to improve the classification results on the problem domain. In particular, Haveai et al. introduced a cascaded, deep neural network architecture that explores image features in the local and global context (Havaei et al., 2017). The proposed architecture uses two-phase training to address the tumor labeling imbalance and the output of the basic CNN model is fed as an additional input into the subsequent CNN model for improved tumor segmentation accuracy. Russakovsky's survey of the scene recognition methodologies includes the side-by-side algorithms comparison which concluded that the CNNs as the multi-stage, hand-tuned feature extractor followed by a discriminant classifier always outperformed the traditional feature coded or the single deep neural network architectures (Russakovsky et al., 2015). The cell image segmentation using small training and validation data was explored using multi-channel feature maps based on multi-path heuristics: the image symmetry coded the localization and the feature context was coded as the contradicting model hypothesis. The output of the proposed U-Net architecture was the segmented and annotated image that could be used as an input to a subsequent work-flow processing stream (Ronneberger et al., 2015).

The key advantage of the CNN is their ability to generalize and learn over large feature set, their invariance to noisy features, a common characteristic of bio-medical image processing, and the ability to infer the features necessary for classification (LeCun et al., 1990, 2004; Jarrett et al., 2009; Lee et al., 2009; Pinto et al., 2009; Turaga et al., 2010). The superior performance of a CNN over traditional (NN, SIFT etc.) architectures is mainly attributed to the ability of the convolutions to recognize the feature invariance despite small changes in position, noise frequency, or rotation (Abdel-Hamid et al., 2013). The drawback of the CNN based architectures is in their reliance on large number of training images needed to automatically prune the stochastically generated convolution filters (kernels), the computational resources needed to implement and train the model, and the lack of knowledge transparency of the final model. The image classification is based on the features extracted using the learned filters, but the filters alone will not explain which image descriptors, structures, or characteristics were used by the final discriminant classifier. The re-application of the learned kernels on the input image may be a useful visualization of what were the filter activations in the images at each CNN's convolution step.

Overall, ML architectures incorporate several different deterministic machines along with a CNN to implement image processing work-flow from the image pre-processing, the feature extraction, to the classification (El-Dahshan et al., 2014; Havaei et al., 2017). Menze et al. designed a hierarchical brain tumor detection algorithm, tested on the BraTS data-set, which used multiple learned models to determine the classification output. The high classification results were achieved by the model ensemble as no single machine achieved the highest classification accuracy on all tasks. Segmentation of the region of interest first is critical to a CNN's classification performance since searching the entire image is computationally prohibitive and the resolution of detected features is too poor to produce high accuracy classifications (Alakwaa et al., 2017). This observation is especially true for 3D composites which have a larger search space compared to 2D images.

A critique of adopting CNNs as the feature extractor compared to many hierarchical or cascaded ML architectures is in the lack of mechanistic explanation used as the basis for the final diagnosis. Instead, the proposed architectures with a near human-grade classification performance feed the outputs of one CNN as the additional inputs to the subsequent processing step or to a classifier, bypassing the transparency rigor (Ronneberger et al., 2015; Russakovsky et al., 2015; Havaei et al., 2017). As a result, the high performing CNN based models are not transferable to other domains or diagnoses, a ML architecture for a new disease diagnosis cannot be heuristically constructed from the building block models, and as the classification accuracy increases so does the ML architecture's complexity but the knowledge transparency decreases. Empirically improving ML classification performance on the scene recognition problem is acceptable, but if a proposed architecture cannot be deconstructed in reverse, from classifier to the original source image manipulation, then its application as a bio-medical decision support tool is likely to fail.

### 5.3. Hierarchical classifications

Incremental algorithms and hierarchical classification (HC) occur in ML architectures where the partial findings (sub-models) are organized into hierarchical relationships to synthesizes a non-trivial classification with multiple decision paths possible (Cesa-Bianchi et al., 2006; Nakano et al., 2017). Breaking-up the final medical diagnosis into a network of partial classifications would improve model transparency by providing contextual, data-set limited, scope defined findings used to infer the final classification.

For example, medical diagnosis of soft tissue pathology from cellular scans can be defined as a HC problem. The cell level pathology is dependent on the local, regional, and global context of the detected features and the final diagnosis is formed as an incremental synthesis from the detected tissue types. However, redefining the classification as a HC problem will require expert knowledge to differentiate the detected tissue features into the predefined classes and organize the sub-models into a hierarchical relationships (Wicker et al., 2007; Santos et al., 2016). The supervised approach to annotate the findings into annotations and relationships used by the subsequent work-flow modes is labor intensive and non-intuitive. Recasting the classification as a HC problem using expert knowledge of the problem domain remains a challenge as it is not often obvious what are the local patterns of interest, how do they relate to each other and how to interpret the local findings in the global context (Wicker et al., 2007).

Hierarchical classification problems have been solved using decision trees for information retrieval from text (Cesa-Bianchi et al., 2006) and neural networks (Santos et al., 2016; Nakano et al., 2017) for successful DNA sequence classification (Wicker et al., 2007) and protein function prediction (Cerri et al., 2016). Properly separating a classification into its hierarchical components also provides the added benefit of reducing the number of possible outcomes, and localizes sub problems making error analysis of the model easier.

### 5.4. Actor-critique models

Actor-critique (AC) ML architectures can provide detailed feedback on how the information structure emerges from raw data inputted into the model. In the case of MRI images, AC architectures can present the features of interest extracted by the model (Joel et al., 2002). AC modeling allows for analysis of the information retrieval work-flow which in turn allows for a side-by-side models comparison as the identification of model's strengths as the correctly extracted information from the images and its weaknesses of missed or incorrectly identified information. Such detail view of a ML architecture goes well beyond the frequency based evaluation of model accuracy, and provides unique opportunities for training and validation.

Fard et al. have shown the robustness of AC ML models to address different types of noise in the planning and habitual control systems (Fard and Trappenberg, 2017). Similar noise characteristics can be found in the MRI scans with low frequency noise features such as the calcium deposits, implants, bone structure or the high frequency noise from the imaging instrumentation error (Kononenko, 2001). More importantly, this type of ML architecture could be used to identify where to critique a learned model after making a classification as well as see why a particular classification error occurred by identifying the specific features that caused the error.

In practice, the AC architecture can reinforce the habitual classifications and learn from previous error, making it robust for handling the imperfect information in the MRI images (Rosenstein and Barto, 2002; Kober et al., 2013; Fard and Trappenberg, 2017). The critique model component will correct the model to meet the target classification if the actor model continuously makes the same classification error. At the same time, the architecture can present where the error took place within the critic network. The actor-critique ML architectures were used to design artificial neural networks to learn the dynamic target location and to provide the model's error back-propagation transparency (Vamvoudakis and Lewis, 2010; Fard and Trappenberg, 2017). The use of AC architecture has not been proposed for other ML algorithms.

### 5.5. Activation maps visualization

Machine learning architectures like ANN and CNN have been considered as “black boxes” due to the difficulty of understanding how the learned model makes its decisions (Bengio et al., 2013; Yosinski et al., 2015). The prevalent use of convolution neural networks, as the feature extractor in many natural scene classification solutions, rely on a large image corpus to learn the convolution filters to identify the local structures in the pixel space that are common across the training set. Visualizing the filter response at each logical unit, in each layer of the CNN allows the semantic explanation of which features were learned across the training images and would allow the medical practitioner to understand the information basis used by the ML architecture in its decision making.

Yosinsi et al. developed a set of tools to show the filter activation maps and the corresponding hierarchy during the classification phase which allows for the visualization of both the low and high level abstract concepts learned by the ML architecture (Yosinski et al., 2015). The tool also allows to visualize the images that contributed to the creation of the learned filters (that correspond to the learned features), how multiple low level features were combined to define more complex features, and how the hierarchy of the activated feature triggered the classification output. For a radiologist, understanding how the low level image characteristics are used for the high level machine reasoning could not only provide the transparency of the information extraction, but could be used to discover new knowledge about the nature of the pathology itself. The visualization tool is specific to the CNN based architectures and does not port to other ML algorithms.

It is important to note that any visualization tool-set is designed specifically to a given ML algorithm, so the ML framework transparency through a visualization is done for each step of the image processing work-flow that corresponds to one ML algorithm.

### 5.6. Visual attention and the regions of interest

If the primary visual features in the MRI images were successfully identified by the radiologists and their diagnostic role is well known and documented, embedding the expert's knowledge into the ML framework construction could improve both the framework's classification accuracy and the knowledge transparency. Improved model performance stems from tuning the model's attention to the visual cues used by the radiologist, which effectively narrows the classification problem for the ML algorithm by providing the preliminary information about detected structures in the image thus offloading the low level feature detection in favor of the high level reasoning.

A common implementation of the visual attention is by initially training a CNN to extract the visual structures from the image, then using these learned, high level, pre-processed structures to guide the low level image processing. In other words, the CNNs use the pixel space to build the feature space as the bottom up heuristics, then the visual attention uses the high-level detected structures as the top-down heat-map of interest. Regions of interest detection using the image pre-processing is implemented in several projects that analyse the natural scene images (Itti et al., 1998; LeCun, 1998; Ioffe and Szegedy, 2015; Ren et al., 2015; Redmon et al., 2016).

Visual attention and identification of the regions of interest allows models to create a bias toward specific structures in the images which could also be used by a radiologist in the feature extraction from the MRI images. One might argue that the ML frameworks that bridge the top-down and the bottom-up model image processing using bias are hindering the generalization process of feature identification which is needed by the classifier. That said, the difficulty of making medical diagnosis from the MRI images and the limited size of the training data-sets could be significantly aided by embedding human intelligence to the model construction.

## 6. The future road map

A machine learning based implementation of an intelligent decision support tool that relies on training and validation data for a medical practitioner must provide high diagnostic accuracy, information transparency, and traceable heuristics used to analyse the source image and conclude the final diagnosis. These technical ML framework requirements are in addition to the usability guidelines outlined in the section 2. Latest trends in the fields of human centered design, human computer interaction (HCI), interactive visualization, and neural computations could provide solutions that have low decision making complexity for a physician, are easily integrated into the existing image processing work-flow and provide new information for a diagnostician.

The human centered design of the ML framework construction has to be focused around the radiologist's domain knowledge, as the key design principles of the intelligent assistant design, model training and evaluation as well as the acceptance or rejection of a final diagnosis. What is the information extracted from the source images can be visualized as the activation maps of the CNN's learned features (Yosinski et al., 2015). A heuristic feedback of the model's information selection and the classification error analysis can be addressed using actor-critique models (Fard and Trappenberg, 2017). Understanding the model's decision making heuristics can be approached by trading the deep model paradigms for hierarchical and stacked model ensembles (Kenji Nakano et al., 2017; Nakano et al., 2017).

The above listed design characteristics are examples of separate research topics, applied to different problem domains that have not been integrated into a single ML architecture (Yosinski et al., 2015; Fard and Trappenberg, 2017; Kenji Nakano et al., 2017; Nakano et al., 2017). The design requirements of a radiology DST is similar to the requirements of other projects such as automated air traffic control, autonomous transportation systems, or autonomous drone technology. Beyond the computational requirements, software engineering challenges and a lack of corpus of training data, we believe the creation of such ML architecture as an intelligent medical assistant is feasible.

### 6.1. Existing open source tools

Over the years, many of the medical image processing research projects produced open source software tools for image pre-processing and complex analysis. Since the tool quality varies, this section summarizes the mature, widely used tools. Table 1 lists both general as well as special purpose tools and frameworks for MRI processing. The table only lists open-source platforms that are free to modify and distribute for non-commercial use. A majority of the tools have source code available which makes them extendable to implement additional functionality or to integrate the existing tools into a larger AI framework. To our knowledge, none of the tools in the list are approved by the U.S. Food and Drug Administration (FDA) for diagnostic purposes.

**Table 1:** A list of current, mature, open-source MRI image processing and ML frameworks as well as the general MRI community portals.

**General**		
NIF	https://neuinfo.org/	Neuroscience Information Framework - a neuroscience community portal
Open fMRI	https://www.openfmri.org/	Community portal for free and open sharing of raw magnetic resonance imaging (MRI) datasets
Open source imaging	http://www.opensourceimaging.org	Developers of open hardware imaging devices and image processing techniques
ANDI	http://adni.loni.usc.edu/	The Alzheimer's disease neuroimaging initiative (ADNI)
**Open source frameworks**		
3D Slicer	https://www.slicer.org	Rich on features, open source, extendable, multi-platform
Brain Voyager	http://www.brainvoyager.com/	Analysis and visualization of structural and functional MRI data combined with EEG or MEG distributed source imaging
Freesufter	http://freesurfer.net/	Both low and high level processing and analyzing of brain MRI images
BrainSuite	http://brainsuite.org/	Software tools for automated processing of brain MRIs
SlicerDMRI		Diffusion MRI Software for Brain Cancer Research
Brain Atlas	http://www.brain-map.org/	A collection of tools for segmentation, registration, and volumetric analysis of the functional brain centers
Fiber Navigator	http://scilus.github.io/fibernavigator/	Fiber visualization and reconstruction toolbox from DTI data
TrackVis	http://trackvis.org/	Fiber visualization and reconstruction toolbox from DTI data
Camino	http://camino.cs.ucl.ac.uk/	DTI image processing and fuber reconstruction
DTI-tk	dti-tk.sourceforge.net	DTI image processing with spatial normalization and atlas construction for white matter morphometry measurements
ANTS	http://stnava.github.io/ANTs/	Advanced Normalization Tools (ANTs)
	http://picsl.upenn.edu/software/ants/	
ITK-Snap	http://www.itksnap.org	Segmentation of 3D structures from medical images using active contours
MITK	http://mitk.org/	A toolbox for development of interactive medical image processing software
Elastix	http://elastix.isi.uu.nl/	A toolbox for rigid and nonrigid registration of images
Gadgetgron	http://gadgetron.github.io/	NIH framework for medical image reconstruction
gpuNUFFT	http://cai2r.net/resources/software	Accelerator gridding library with Matlab interface
BART	http://mrirecon.github.io/bart/	Berkeley Advanced Reconstruction Toolbox
	https://lists.eecs.berkeley.edu/sympa/info/mrirecon	
Pulseq	http://pulseq.github.io/	Open-source pulse sequences
JEMRIS	http://www.jemris.org/	Multi-purpose MRI sequence development and simulation environment
MARIE	https://github.com/thanospol/MARIE	Magnetic Resonance Integral Equation Suite
NiftyRec	https://sourceforge.net/projects/niftyrec/	Reconstruction library
**General MRI, imaging or ML libraries**		
GIMIAS	http://www.gimias.org/	A workflow-oriented environment for solving advanced, biomedical image computing and individualized simulation problems
SPM	https://www.fil.ion.ucl.ac.uk/spm/	The analysis of brain imaging data sequences using Statistical Parametric Mapping as an assessment of spatially extended statistical processes used to test hypotheses about functional imaging data
FSL	https://fsl.fmrib.ox.ac.uk	A collection of analysis tools for FMRI, MRI and DTI brain imaging data
PyBrain	http://pybrain.org/	Reinforcement Learning, Artificial Intelligence and Neural Network Library
TensorFlow:	http://tensorflow.org	ML and AI frameworks
Caffe	http://caffe.berkeleyvision.org/	A deep ML framework
PyMVPA	http://www.pymvpa.org/	Statistical learning analysis platform
Weka	https://www.cs.waikato.ac.nz/ml/weka/	Data mining platform
Shogun	http://www.shogun-toolbox.org/	Machine learning framework
SciKit Learn	http://scikit-learn.org	Scientific computation libraries
PRoNTo	http://www.mlnl.cs.ucl.ac.uk/pronto/	Machine learning framework

*The table includes the standalone, purpose built packages as well as the extendable frameworks for the MRI image processing and machine learning*.

## 7. The summary and discussion

In recent years, the fields of computer vision, machine learning, artificial intelligence and imaging robotics have experienced a boom due to the availability of large data sets, engineering of intelligent algorithms, and the support of high throughput computational platforms (LeCun et al., 2015; Menze et al., 2015; Alakwaa et al., 2017; Havaei et al., 2017). As a result, the performance of the AI algorithms analyzing the unstructured information in the images is approaching or exceeding human performance on a limited problem domain such as the natural scene classification or object detection. These trends present a unique opportunity of applying and integrating currently developed methods to the largely unexplored problem of diagnostic analysis of the brain pathologies from the MRI images.

Analysis of the human brain MRI based image sets also presents a unique set of challenges that must be addressed in order to build the decision support tools that can be easily integrated into the existing image-based diagnostic work-flow used by the radiologists and other medical professionals. The tool's goal is to augment the process of a human decision making rather than replacing a man by a machine. The tools must eliminate the type 2 diagnostic classification error (missing a diagnosis). An interactive visualization and alternative construction of the ML architectures will provide a transparent and traceable analysis of what is the useful image information and how did the ML framework arrived at the final diagnosis. This will also allow for a physician-in-the-loop diagnostic verification. Finally, the tool has to integrate into the diagnostician's decision making stream with a minimum added complexity. These human centered engineering requirements are in addition to the technical challenges unique to the analysis of the MRI images that include detection of diffused features, processing image signal in the presence of both high and low frequency noise, feature validation across multiple scopes (local, regional and global), encoding the feature-to-feature relationship in 3D, and designing custom hardware and ML framework architectures to support the computational needs for processing of the 2D and 3D MRI data sets.

To address these challenges, the radiologist's expert knowledge has to be integrated in each step of the image processing work-flow which is only possible using an iterative human centered design with a level playing field between the tool's engineers and the medical professionals. The technical approach to address the knowledge transparency and the decision making explanation is likely to trade a single, deep computational paradigm for a machine learning framework that consists of a hierarchy of stacked sub-models that extract and process meaningful information in the forward direction with the ability to trace-back the framework's decision making.

The fundamental requirement to construct a high diagnostic accuracy ML framework is an availability of a unbiased, large, diverse, correctly annotated data-set. Currently, the ML community does not have an access to such data-sets. The training and validation corpus has to include a hard to detect tissue anomalies. The data-set has to be annotated and cross-validated by the radiologists with expert knowledge of the brain pathologies. The annotation labels have to provide ground-truths about the imaged tissue on all analytical scales: local, region and global. The resulting image collections will provide training heuristics for the ML algorithms to address the hard questions of early disease detection, monitoring brain's recovery progress, and aiding in the disease diagnosis that cannot be done by a naked eye. The data-set should consist of images taken by the latest imagine robotics with high resolution and low slice thickness. That said, even the largest data sets will be considered “small-data”, in terms of the number of samples, in comparison to the training images used to train other computer vision neural networks, and therefore expert guidance in model development and feature extraction could be used in order to achieve parity with the state of the art in other computer vision fields with much larger datasets.

## Author contributions

All authors listed have made a substantial, direct and intellectual contribution to the work, and approved it for publication. All authors equally contributed in preparation of this manuscript.

### Conflict of interest statement

The authors declare that the research was conducted in the absence of any commercial or financial relationships that could be construed as a potential conflict of interest.

## Footnotes

^1^Applications of medical diagnosis using MRI images beyond those studied in this paper include alcoholism, dementia, Alzheimer's disease, stroke, concussion, bleeding, trauma, schizophrenia, bipolar disorder, progressive multifocal, leukoencephalopathy, and multiple sclerosis.

^2^https://www.slicer.org

^3^http://braintumorsegmentation.org/

^4^The final results of 2017 competition were not available at the time of this publication.
